# Predictors of Neonatal mortality in Neonatal intensive care unit at referral Hospital in Southern Ethiopia: a retrospective cohort study

**DOI:** 10.1186/s12884-019-2227-5

**Published:** 2019-02-28

**Authors:** Tujare Tunta Orsido, Netsanet Abera Asseffa, Tezera Moshago Berheto

**Affiliations:** 1 Wolaita Sodo University Teaching and Referral Hospital, Wolaita Sodo, Ethiopia; 2 School of Public Health, Wolaita Sodo University, 138 Wolaita Sodo, Ethiopia

**Keywords:** Neonatal mortality, Neonatal intensive care unit, Ethiopia

## Abstract

**Background:**

The first one month of life; the neonatal period is the most risky time for child survival. In Ethiopia, neonatal mortality is unacceptably high, and the trend in reduction is slower as compared to infant and child mortality. The magnitude and associated factors of neonatal mortality in a tertiary care facility were not well documented. Therefore, the aim of this study was to determine neonatal mortality and predictors among neonates admitted to neonatal intensive care unit of Wolaita Sodo University Teaching and Referral Hospital, South Ethiopia.

**Methods:**

A retrospective cohort study design was done among neonates admitted to neonatal intensive care unit of a University Teaching and Referral Hospital from 2015 to 2017. Data were collected using data extraction checklist from the medical registry. The main outcome was the occurrence of death within the first four weeks. The survival time was calculated in days between the date of admission and the date of death. Kaplan-Meier survival was used to depict the pattern of death in 28 days and Cox-Proportional model was used to identify the predictors of the neonatal mortality.

**Results:**

A total of 964 neonates which contributed to 5889 neonates-days were included in the study. There were 159 neonatal deaths during the follow-up time. Overall, the neonatal mortality incidence was 27 per 1000 neonates-days. Predictors of neonatal mortality were: multiple birth, mothers who did not attend antenatal care visits, neonates born by cesarean section, not initiated breast feeding within 1 h of birth, neonates resuscitated, hyaline membrane disease and perinatal asphyxia.

**Conclusion:**

Neonatal mortality at neonatal intensive care unit was high. Managing neonatal complications, initiating breast feeding within 1 h of birth, promoting antenatal care visits, improving quality of services and ensuring continuum of care are recommended to increase survival of neonates.

## Background

The first one month of life; the neonatal period is the most risky time for child survival. The world health organization reported 2.6 million deaths or roughly 46% of all under-five deaths occurred in 2016, up from 40% in 1990; nevertheless, neonatal mortality fell by 49% from 37 deaths per 1000 live births in 1990 to 19 in 2016 [1–3]. Ethiopia is one of the ten countries which accounts to two-third of global neonatal death and also among the six countries accounting half of global under five deaths [3, 4]. Although developing countries have been given highest priority to achieve the global target of less than ten deaths per 1000 live births by 2035; it is projected that the sub-Saharan Africa will have 33% of births and 60% of deaths in 2030, compared to 25% births and 50% deaths in 2013 [5, 6].

The world has made substantial progress in reducing under five deaths from causes such as diarrhea, pneumonia and measles (3.3 million fewer deaths in 2013 versus 2000). Nevertheless, congenital, preterm, neonatal sepsis and injury remain leading causes of death [6]. Several previous studies have identified a number of predictors that lead to neonatal deaths such as preterm birth (28%), sepsis (26%) and asphyxia (23%). In developing countries, sepsis is the most common cause of neonatal mortality and is probably responsible for 30–50% of the total neonatal deaths each year [1, 7, 8].

Literatures showed that neonatal mortality is the outcome of complex relationship between neonatal, maternal and health care-related factors. In spite of many efforts by the government and other partners, non-significant decline has been achieved in the last 20 years [9, 10]. Between 1990 and 2015, the Ethiopia’s neonatal mortality dropped from 63 to 23 per 1000 live births with annual fall of 2.2% [11]. According to EDHS (2016), Ethiopia is among the countries with the highest neonatal mortality with the rate of 29 deaths per 1000 live births. It is worth noting that 42% of the under-5 mortality in Ethiopia is attributable to neonatal deaths [12, 13]. Identifying and understanding factors such as prenatal care, mode of delivery and labour can lead to reduction in neonatal mortality. Studies undertaken to assess the influence of these variables on neonatal mortality may have used descriptive designs which means fall in short to explain association [9]. It is useful to identify risk factors that predict early infant mortality, especially those that have the potential for intervention with modest resource [14].

The causes of neonatal mortality are not well documented in Ethiopia mainly at institutional level including this study area, but there are few studies that reported causes such as sepsis, asphyxia, preterm birth, congenital malformations [13]. With identification of risk factors, it is possible to prevent, particularly aiming the improvement of newborn children care. Here, the neonatal intensive care units (NICUs) is one of the most effective tools to reduce country’s newborn mortality [15]. Therefore, this study assessed the predictors of neonatal mortality among neonates admitted to neonatal intensive care unit of Wolaita Sodo University Teaching and Referral hospital, southern Ethiopia.

## Methods

### Study setting and period

This study was done in Wolaita Sodo University Teaching and referral hospital, department of pediatrics from October 11–November 10, 2017. The university hospital serves more than three million people in Wolaita and neighboring zones. The pediatrics department had four major wings: pediatric Emergency admission unit, Neonatal Intensive Care Unit (NICU), pediatric surgical admission ward and pediatric medical admission ward. There were a total of 75 patient beds in the four wings; of which Neonatal ICU had 20 beds. The ICU was established in June, 2014 [16].

### Study design

A retrospective cohort study design was conducted among admitted neonates.

### Source population

All medical records of neonates admitted between October 2015 and October 2017 were included.

### Study population

All records fulfilling the inclusion criteria from the source population.

### Sample size determination

The sample size was calculated by using Open Epi-info version 3.03. The following considerations were made for the cohort study neonatal death was considered as an outcome variable and from predictors, single birth compared with multiple births. We assumed multiple birth as exposed and single birth as unexposed considering a 1.9 hazard-ratio [17]; 95% level of confidence and 80% power, the minimum sample size computed was 506. However, to improve the precision of the test the final sample size was 964 by including all neonates admitted to the NICU in the study periods.

### Inclusion criteria

All neonates registered on neonatal registry book from October 2015 to October 2017 in Neonatal ICU of the teaching hospital were included.

### Exclusion criteria

Neonatal medical record with incomplete information, age greater than 28 days and revisits were excluded from the study.

### Sampling techniques

Neonatal medical record done from October 2015 to October 2017 in NICU of WSUTRH was retrieved by using data extraction checklist which was adopted from national neonatal registration book.

### Measurement

Times- to- event, the event of interest was neonatal death.

### Exposure variables

Maternal and Neonatal related factors: ANC follow-up, parity, gravidity, mode of delivery gestational age, birth weight, age of neonate at discharge, sex of neonate and temperature of neonate at admission. Neonatal illness during admission: respiratory distress, perinatal asphyxia, sepsis, congenital malformation, hyaline membrane disease, meconium aspirated syndrome. Care/service related factors: newborn resuscitated, initiation of breast feeding, treatment given and length of stay.

### Data collection procedure and quality assurance

Data was abstracted by three trained nurses who had diploma and experience in the setting. The overall data collection process was supervised by trained BSc nurse. She was trained on how to revise the registry and then abstract secondary data from medical records. The principal investigator used to supervise by using data extraction checklist.

### Data management and analysis procedures

Death of neonate was the event of interest which was coded as “1” for failure and “0” for censored. Time-to-event was calculated by subtracting the date of admission from the date of event. Data was entered into Epi-Data version 4.0.1.44 and exported to SPSS 20 for the analysis. The two dataset entered were validated for consistency and 7% error was corrected from the original questionnaire. Frequencies, percentages and rates were computed. Analysis was carried out for explanatory data. Moreover, multi-colinearity was checked using correlation coefficient (r) and value of *r* <  0.7 was used as a cutoff point when two variables have bivariate correlation. There was bivariate correlaton between parity and gravidity, gestational age and prematurity with *r* = 0.84 and *r* = 0.99 were found respectively. Mean and standard deviation were computed for follow-up time and age of the cohort. Cox-Proportional hazard model was used to determine factors associated with the survival time of neonates admitted in the NICU.

Kaplan Meier curves were used to estimate survival time. Log Rank test was used to look statistical differences between the categories of variables. Statistical significance was declared at *p*-value < 0.05. A summary statistics of proportions including hazard ratio and 95% confidence intervals as used. Screening of predictors of neonatal mortality was done by using bivariate Cox regression for each variable one at a time. Then, those variables with *p* <  0.25 during the bivariate Cox regression analysis were taken as a candidate variables for multivariate Cox regression model to control possible confounders.

## Results

During the two years of observation, 1026 neonates were admitted at WSUTRH Neonatal ICU, followed for 5889 neonate event free days. The records of 62 neonates were excluded because of predetermined exclusion criteria; 39 (3.8%) incompleteness, 15(1.5%) age above 28 days and 11(1.1%) revisits. Therefore, the present study included records of 964 neonates with optimally complete information. The mean (±SD) length of stay at NICU was 6.1(5.1) days. Of the 964 neonates 752(78%) were discharged when their condition improved, 159(16.5%) died, 29(3%) went against medical advice, 21(2.2%) referred to another hospital after their primary reason for admission was managed in the unit and 2(0.3%) no change (those with terminal conditions).

### Neonatal and maternal characteristics

Out of 964, 574(59.5) were male neonates making the male to female ratio 1.5:1. The mean (±SD) age of neonate at discharge was 6.5(5.4) days. More than half 561(58%) were age less than 7 days. Majority 752(78%) of neonates were term. Among all admitted neonates 528(54.8%) were born from multiparous mothers and 452(46.9%) neonates were born from mothers with Antenatal Care four and above visits (Table 1).

**Table 1:** Frequency of neonatal and maternal factors of neonates admitted to WSUTRH, South Ethiopia, from 2015 to 2017

Variables	Categories	Frequency	Percentage
Mode of delivery	SVD	602	62.4
	Assisted vaginal	161	16.7
	C/S	201	20.9
Birth weight of neonates	< 2500 g	333	34.5
	= > 2500 g	631	65.5
Birth level of neonates	Single	821	85.2
	Multiple	143	14.8
Temperature of neonate at admission	< 35.5 °C	323	33.5
	35.5–37.5 °C	579	60.1
	> 37.5 °C	62	6.4

### Neonatal illness and service related factors

**Fig. 1:**
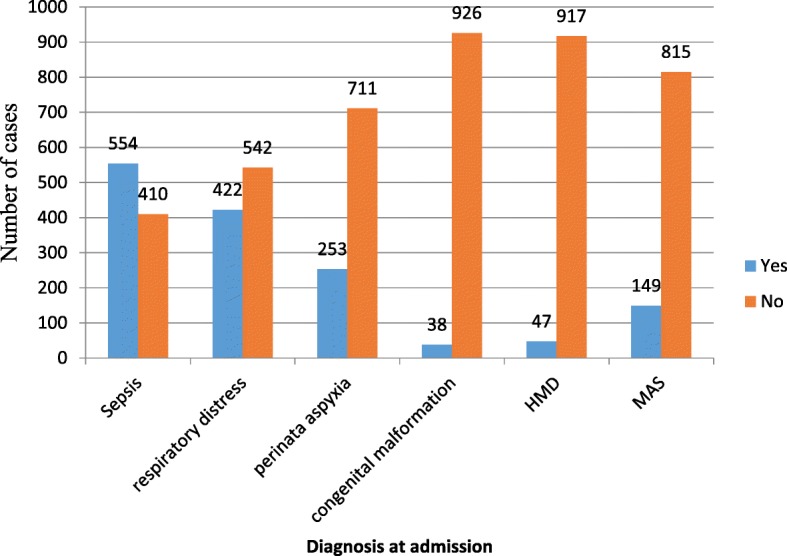
Neonatal illness identified at admission in NICU of WSUTRH, South Ethiopia, 2015–2017

The most common problem identified at admission was neonatal sepsis 554(57.5%) followed by respiratory distress 422(43.8%) while the least common was congenital malformation 38(3.9%) (Fig. 1). Concerning breast feeding, 519(53.8%) neonates were breast fed within 1 hour of birth.

### Neonatal outcome

**Fig. 2:**
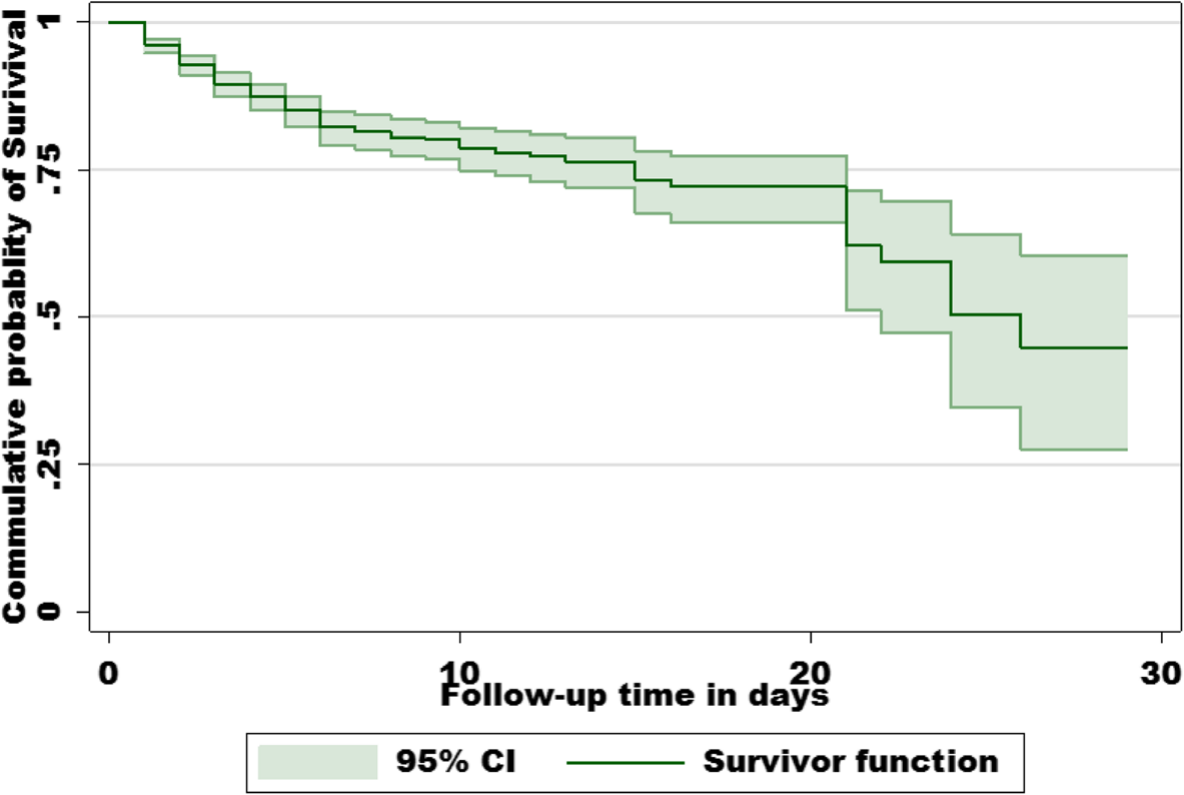
K-M survival estimate of neonates hospitalized at NICU of WSUTRH South Ethiopia 2015–2017

During the study period, a total of 16.5% (*n* = 159) deaths of neonates were recorded. Of the 159 neonatal deaths, 132(83%) or 77 neonatal deaths per 1000 neonate-days were early neonatal mortality (0-6 days) and 27(17%) or six neonatal deaths per 1000 neonate-days were late neonatal deaths (7–28 days) (Fig. 2). The overall incidence of neonatal mortality was 27 neonatal deaths per 1000 person- days (95% CI: 23.1, 31.5).

There was a lower level of late neonatal mortality rate compared to early neonatal mortality rate. It shows a significant decline in mortality after neonates survive the first 7 days of life in the observation period (Fig. 2). In the study out of the total admission, male deaths 112(11.6%) outnumbered female deaths 47(4.9%). Hyaline membrane disease 31(66%), perinatal asphyxia 58(22%) and congenital malformation 2(5.3%) were identified as the leading causes of death during admission.

### Predictors of neonatal mortality in NICU of WSUTRH, South Ethiopia, 2015–2017

The Variables that were found to have association with the death of neonates during bivariate Cox regression were: sex of neonate female, birth weight of neonate < 2500 g, gestational age of neonate preterm, multiple birth (Table 2).

**Table 2:** Bivariate and Multivariate Cox-proportional hazard regression for predictors of neonatal mortality among neonates admitted to NICU of WSUTRH, South Ethiopia, 2015–2017

Variables	Outcomes		p-value	Crude HR 95%CI	Adjusted HR 95%CI
	event	censored			
Sex of neonate					
male	112	462	1	1	1
Female	47	343	0.003	0.59 [0.42–0.84]	0.91 [0.64–1.30]
Birth weight					
< 2500 g	104	229	<	3.41 [2.46–4.73]	1.32 [0.78–2.25]
> = 2500 g	55	576	< 0.001 1	1	1
GA					
term	75	677	1	1	1
Preterm	84	128	< 0.001	3.60 [2.63–4.93]	0.92 [0.48–1.78]
Birth level					
multiple	69	74	<	4.43 [3.24–6.07]	1.80 [1.10–2.94]^*^
Single	90	731	0.001 1	1	1
ANC follow-up					
> 4	19	433	1	1	1
1–3	60	286	< 0.001	4.06 [2.42–6.81]	2.76 [1.62–4.71]
None	80	86	< 0.001	12.96 [7.86–21.39]	6.02 [3.52–10.27]**
Parity					
primipareous	86	269	< 0.001	2.11 [1.58–2.96]	1.19 [0.86–1.66]
Multipareous	73	536	1	1	1
Mode of delivery					
SVD	136	466	1	1	1
SVD	10	151	< 0.001	0.28 [0.15–0.53]	0.39 [0.20–0.75]
C/S	13	188	< 0.001	0.26 [0.15–0.46]	0.34 [0.19–0.61]^**^
Suspected sepsis					
Yes	106	448	1	1	1
No	53	357	0.021	0.68 [0.49–0.94]	0.83 [0.54–1.28]
Respiratory distress					
Yes	74	348	> 0.99	0.0.98 [0.73–1.36]	–
No	85	457	1	1	
PNA					
Yes	58	195	0.001	1.73 [1.25–2.39]	1.81 [1.24–2.63]^*^
No	101	610	1	1	1
Congenital Malformation					
Yes	2	36	0.105	0.32 [0.08–1.28]	
No	157	769	1	1	–
HMD					
Yes	31	16	< 0.001	4.60 [3.10–6.82]	2.04 [1.16–3.59]*
No	128	789	1	1	
MAS					
Yes	20	129	0.217	0.74 [0.47–1.19]	
No	139	676	1	1	–
Temperature of neonate at admission					
35.5–37.5	42	537	1	1	1
< 35.5	110	213	< 0.001	4.75 [3.33–6.78]	1.58 [1.06–2.34]*
> 37.5	7	55	0.343	1.47 [0.66–3.28]	1.58 [0.70–3.59]
Neonate resuscitated					
Yes	122	327	< 0.001	4.49 [3.10–6.49]	2.28 [1.54–3.38]**
No	37	478	1	1	1
Breast feeding initiated					
within 1 h	22	497	1	1	1
after 1 h	137	308	< 0.001	7.42 [4.73–11.64]	2.62 [1.60–4.30]**
Treated by oxygen					
Yes	139	477	1	1	1
No	20	328	< 0.001	0.27 [0.17–0.43]	0.65 [0.39–1.06]
Treated by KMC					
Yes	53	108	< 0.001	2.22 [1.59–3.09]	0.81 [0.52–1.28]
No	106	697	1	1	–
Treated by antibiotics					
Yes	145	720	1	1	–
No	14	14	0.93	0.97 [0.63–1.69]	
Treated by glucose					
Yes	54	268	0.97	1.01 [0.72–1.40]	
No	105	697	1	1	–

**p < 0.001, *p < 0.05, CHR-crude hazard ratio, AHR-adjusted hazard ratio and CI-confidence interval. n was the same for the crude and adjusted models

**Fig. 3:**
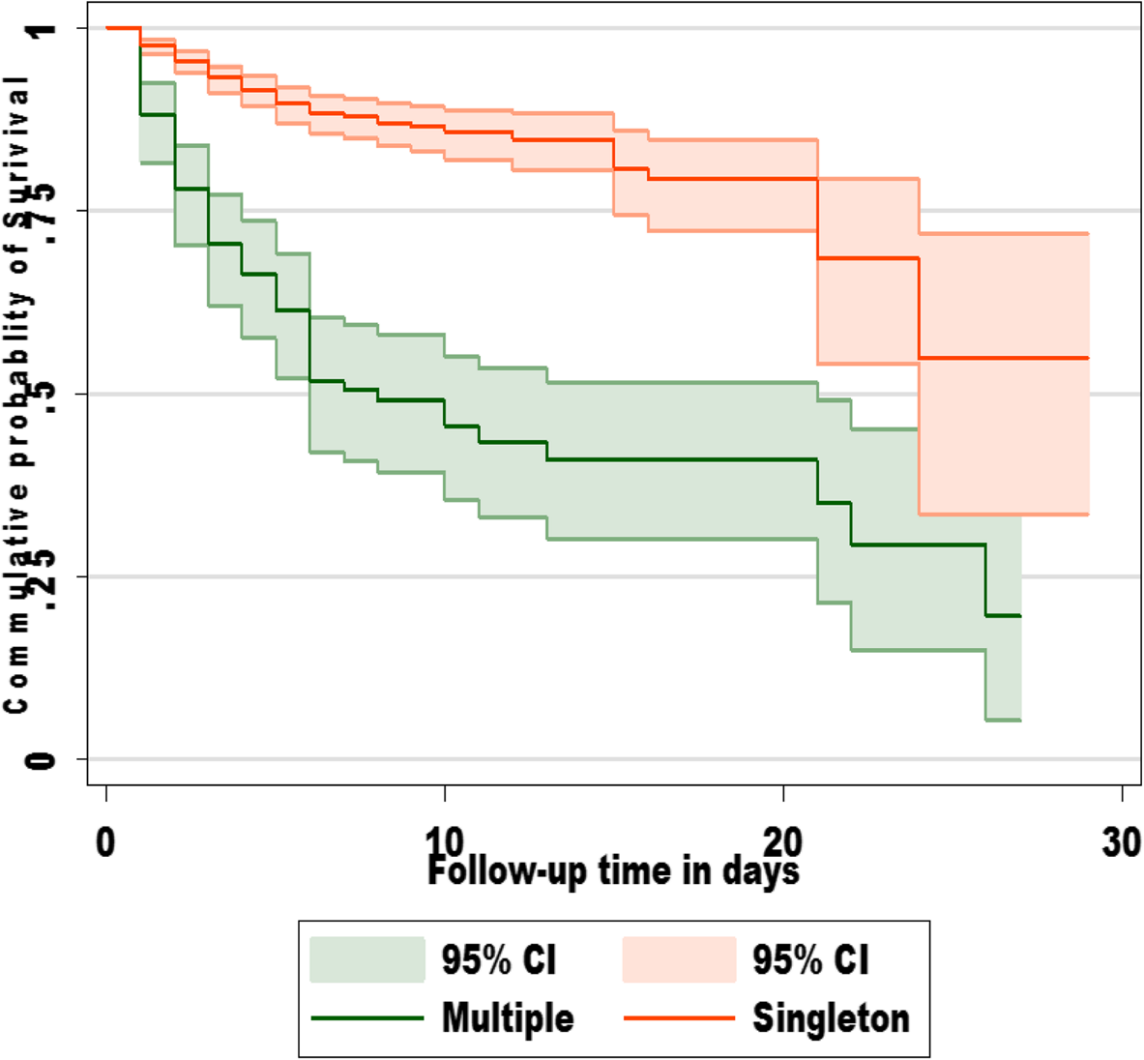
K-M survival estimates of neonates by singleton status at NICU of WSUTRH 2015–2017

Multivariate Cox regression revealed that neonates of multiple births had 1.8 times higher risk of death than singleton neonates, AHR [95%CI] 1.8[1.10–2.94] (Fig. 3). Neonates born from mothers who did not attend ANC visits during their pregnancy were six times at higher risk of death than neonates born from mothers who had ANC visits 4 and above AHR [95%CI] 6.02[3.52–10.27]. Neonates born by cesarean section had a 66% lower risk of death as compared with spontaneous vaginal delivery (SVD) AHR [95%CI] 0.34[0.19–0.61]. Neonates admitted with the problem hyaline membrane disease (HMD) and perinatal asphyxia (PNA) had two times and 1.8 times higher risk of death than their counterparts [95%CI] 2.04[1.16–3.59] and 1.81[1.24–2.63] respectively.

Neonates who had temperature of < 35.5 °C at admission had 1.6 times higher risk of death than neonates temperature at admission 35.5–37.5 °C AHR [95%CI] 1.58[1.06–2.34]. Neonates who were resuscitated had two times higher risk of death than neonates who were not resuscitated AHR [95%CI] 2.28[1.54–3.38] and those who were not breast fed within 1 h of birth had 2.6 times higher risk of death than their counterpart. AHR [95%CI: 2.62[1.60–4.30] (Table 2).

## Discussion

This study aimed to determine incidence rate of neonatal mortality and its predictors among neonates admitted to Neonatal ICU of WSUTRH. Accordingly, the incidence rate of neonatal mortality was 27 deaths per 1000 neonate-days [95%CI: 23.1–31.5]. This finding is higher than retrospective studies conducted in NICU in the northern Ethiopia and other studies done in similar settings [15, 18, 19]. The reason might be that some of previous studies used data from only delivery room and others used from both delivery and neonatal intensive care units also might be related with the quality of service provided, while this study used data of those neonates only admitted to neonatal intensive care unit. Moreover, neonates transferred to Neonatal ICU for admission might have r risk of death as compared to those stayed with their mother in the delivery room [20].

This finding is lower than the study conducted using 5 years data (2001–2005) in NICU in the central Ethiopia where mortality rate was 233 per 1000 live births. This inconsistency might be related to the fact that this study used only perinatal period data-the first week of life. Moreover, it might be related to time difference as the government and partners have implemented several interventions in the last one and half decade. Moreover, the rate was lower than other studies conducted in Pakistan, Burkina Faso and Iran [21–23]. The difference could be that sources of data; these studies collected data from mothers who had different obstetric complications whereas this study did not consider that. And the other might be difference on study design. Neonates born from mothers who have complications during birth may have a higher chance of death. This study considered neonates admitted to NICU regardless of maternal status. This study is comparable with a trend analysis study done in Ghana [24].

In this study multiple births were at increased risk of death compared to neonate born singleton. This study is consistent with other studies done in tertiary hospitals in Ethiopia [25–27]. The multiple born neonates are at increased risk of prematurity and low birth weight, which may makes them susceptible to the risk of infection.

The findings of this revealed that those neonates born from mothers who had no ANC visit had six times higher risk of neonatal mortality as compared to those who had four and above ANC visits. This is similar to previous studies prospective studies done in Ethiopia [8, 9, 28]. The likely explanation could be that having ANC visit during pregnancy indirectly saves the lives of mothers and babies by promoting and establishing good health before childbirth and the early postnatal period [29]. Moreover, maternal health and health care are important determinants of neonatal survival. Based on the fact that neonatal outcomes are affected by maternal health throughout the pregnancy period; as result neonate born from mothers who had no ANC visits may have increased chance to be born with poor neonatal outcome.

This study revealed that mothers delivered by cesarean section had 66% protective effect on risk of neonatal mortality compared to SVD. This finding is comparable with studies conducted in Northwest parts of Ethiopia [19, 30, 31]. The possible explanation might be timely decision rather than waiting for vaginal delivery, delivering by C/S can reduce the risk of death by decreasing the complications due to prolonged labour. This result is contrary with study conducted at Pakistan [7] whereby C/S had increased neonatal mortality which could have resulted from decision making prolonged labour and poor quality of operation procedure. Moreover, it might be related to the outcome of C/S which might hamper breast feeding practice. In this study, neonatal illnesses were one of the independent predictors for neonatal mortality. The causes of neonatal deaths were hyaline membrane disease and perinatal asphyxia. This is comparable with other studies conducted in Ethiopia and Ghana [4, 24, 25]. The possible reason is that in this study SVD outnumbered other mode of deliveries. It is known that SVD might happen after prolonged labour, hence it might led to respiratory complication.

The current study revealed that initiating breast feeding after 1 hour of birth has higher risk of neonatal mortality compared their counterpart. The finding is comparable with a prospective study done in Ethiopia [25]. The timely initiation of breast feeding is essential to reduce newborn death [20]. However, in current study, initiating breast feeding within 1 hour of birth seems inadequately practiced and promoted. It is worth to note that neonates who are sick might not be able to suck breast milk as compared to healthier neonates.

The current study showed that resuscitating neonates in NICU has increased risk of death compared to their counterpart. This may be related to the fact that neonates being transferred to NICU might be at increased risk of neonatal complication or it might be related with using improper procedure to resuscitate and also may be the commitment of care provider. In addition, it might be related to the fact the hospital had comprehensive nurses who have not had adequate clinical attachment and/or experience on neonatal resuscitation. No other studies showed such kind of association.

The limitations of the study is that there is a potential to miss neonatal deaths particularly those registries considered as incomplete which would underestimate the overall neonatal mortality. The other limitation is that some important predictors of neonatal mortality were not explored because of missing data and lack of information.

## Conclusion

There was high incidence of neonatal mortality in Wolaita Sodo University teaching and referral Hospital, which seekuns due attention in order to meet the goal of child survival in Ethiopia- reducing from 28 per 1000 in 2013 to 11 per 1000 live births in 2020. Multiple births, neonates born from mothers who did not attend ANC visit, neonates delivered by SVD, initiating breast feeding after 1 hour of birth, neonates with hyaline membrane disease and Perinatal Asphyxia, and resuscitating were significant predictors of neonatal mortality.

The neonatal intensive care unit should also work on early diagnosis and appropriate management at admission; and continuous care. The maternity ward should work on improvement within facility and others of catchment institutions to increase ANC visits, to improve obstetrics care services and early identification of severely asphyxiated neonates. Moreover, the hospital management should work to improve service provision at maternity ward and neonatal intensive care unit. Further studies using strong designs like prospective study should be conducted to explore more maternal, neonatal and care related factors.

## Abbreviations

ANC: Antenatal care
AOR: Adjusted odds ratio
CI: Confidence interval
EDHS: Ethiopian demographic and health survey
ETB: Ethiopian birr
HDSS: Health and demographic surveillance system
HMD: Hyaline membrane disease
HR: Hazard ratio
IMR: Infant mortality rate
KMC: Kangaroo mother care, K-M- Kaplan Meier
LBW: Low birth weight
MAS: Meconium aspirated disease
NGO: Nongovernmental Organization
NICU: Neonatal Intensive Care Unit
NMR: Neonatal mortality rate
OR: Odds ratio
PNA: Perinatal asphyxia
SD: Standard deviation
SDG: Sustainable developmental goal
SPSS: Software package of social science
SSA: Sub-Saharan Africa
WHO: World Health Organization
